# Incidence of convulsive epilepsy in a rural area in Kenya

**DOI:** 10.1111/epi.12236

**Published:** 2013-06-10

**Authors:** Anthony K. Ngugi, Christian Bottomley, J. Anthony G. Scott, Victor Mung'ala–Odera, Evasius Bauni, Josemir W. Sander, Immo Kleinschmidt, Charles R. Newton

**Affiliations:** ^1^ KEMRI/Wellcome Trust Research ProgrammeCentre for Geographic Medicine Research (Coast)KilifiKenya; ^2^ Department of Infectious Disease EpidemiologyFaculty of Epidemiology and Population HealthLondon School of Hygiene and Tropical MedicineLondonUnited Kingdom; ^3^ Studies of Epidemiology of Epilepsy in Demographic Surveillance Systems (SEEDS)–INDEPTH NetworkAccraGhana; ^4^ Health Challenges and Systems ProgrammeAfrican Population and Health Research Centre (APHRC)NairobiKenya; ^5^ MRC Tropical Epidemiology GroupFaculty of Epidemiology and Population HealthLondon School of Hygiene and Tropical MedicineLondonUnited Kingdom; ^6^ Nuffield Department of MedicineUniversity of OxfordOxfordUnited Kingdom; ^7^ INDEPTH NetworkAccraGhana; ^8^ Department of Clinical and Experimental EpilepsyUCL Institute of NeurologyLondonUnited Kingdom; ^9^ SEIN – Epilepsy Institutes in the Netherlands FoundationHeemstedeThe Netherlands; ^10^ Neurosciences UnitUCL Institute of Child HealthLondonUnited Kingdom; ^11^ Clinical Research UnitLondon School of Hygiene and Tropical MedicineLondonUnited Kingdom; ^12^ Department of PsychiatryUniversity of OxfordOxfordUnited Kingdom

**Keywords:** Epilepsy, Convulsive epilepsy, Incidence, Kenya

## Abstract

**Purpose:**

There are only a few studies of incidence of epilepsy in low and middle income countries (LMICs). These are often small and conducted in specific age groups or areas where the prevalence of risk factors is high; therefore, these studies are not representative of the wider populations. We determined the incidence of convulsive epilepsy (CE) in a large rural population in Kenya.

**Methods:**

We conducted two cross‐sectional surveys 5 years apart within a health and demographic surveillance system. Initially we identified residents without epilepsy who were then reexamined in the follow‐up survey to determine incidence. We estimated the overall incidence of CE and incidence by age‐group, sex, and by administrative location. Estimates were adjusted for attrition during case identification and for the sensitivity of the screening method.

**Key Findings:**

In a cohort of 151,408 people, 194 developed CE over the 5 years. The minimum crude incidence rate was 37.6/100,000 persons per year (95% confidence interval (CI) 32.7–43.3) and adjusted for loss to follow‐up, and the sensitivity of the survey methodology was 77.0/100,000 persons per year (95% CI 67.7–87.4). Incidence was highest in children 6–12 years (96.1/100,000 persons per year; 95% CI 78.4–117.9), and was lowest in the 29–49 year age group (37.4/100,000 persons per year; 95% CI 25.7–54.7).

**Significance:**

We estimated a high incidence of convulsive epilepsy in this population. Incidence was highest early and late in life, suggesting that preventive interventions should target exposures that occur in these age groups. Incidence of focal epilepsy was more than twice that of generalized epilepsy, suggesting that etiologies that are amenable to intervention were most important in this population. It is likely that incidence is underestimated because of the early mortality of incident cases.

Epilepsy is a common neurologic condition and an important cause of disability and mortality (Carpio et al., 2005; Forsgen et al., 2005; Kaiser et al., 1998), accounting for 1% of days lost to illness globally (Leonardi &Ustan, 2002). It is estimated to affect around 70 million people in the world (Ngugi et al., 2010), of whom 80% live in the poorer regions of the world (Newton & Garcia, 2012). These estimates are based on life‐time prevalence data, which may underestimate the burden, since epilepsy is associated with considerable mortality early after the onset of seizures and it may spontaneously remit (Sander, 1990; Kwan & Sander, 2004; Carpio et al., 2005; Diop et al., 2005), leading to poor recall of seizure events (Sillanpaa et al., 2008). Incidence is not affected by either mortality or remission and may provide a more accurate assessment of the burden of epilepsy.

There are, however, only a few estimates of incidence in low and middle income countries (LMICs) (Ngugi et al., 2011). This is due mainly to lack of health facility data to identify incident cases and the high cost of continuous surveillance of population‐based cohorts required for accurate incidence estimates.

We estimated the incidence of convulsive epilepsy (CE) in a large population‐based cohort in a rural area of Kenya over a 5‐year period. We targeted CE, as opposed to all epilepsies, because convulsive epilepsies are easier to identify in cross‐sectional settings and thus do not require specialists, thereby reducing cost and enabling us to survey a large population efficiently. Furthermore, we compared the prevalence of CE 5 years apart to determine if the reduction in infectious diseases such as malaria and meningitis had changed the prevalence.

## Methods

The study consisted of two cross‐sectional surveys, conducted 5 years apart, nested in a demographic surveillance system. In the first survey we determined the active convulsive epilepsy (ACE) status of residents to identify a cohort of individuals without epilepsy; in the second survey we reexamined this cohort to determine the number who had developed ACE during the intervening period. The incidence of epilepsy was then calculated by dividing the number of incident cases by the person years of observation among cohort members (reidentified in the second survey).

### Study setting and study population

We conducted the study within the Kilifi Health and Demographic Surveillance System (KHDSS – http://www.kemri-wellcome.org/khdss/), which covers an area of 891 km^2^, in Kilifi District, one of the poorest districts in Kenya comprising a population of 233,881 persons in 2008 living in 25,526 homesteads (Scott et al., 2012). The KHDSS area was mapped using global positioning system (GPS) receivers, and digital maps derived from these data were used to locate homesteads and follow study participants. Community‐based field workers conduct reenumeration and vital status registration to update the population registers by visiting every homestead two or three times per year. The population is served by the Kilifi District Hospital (KDH), which is located at the center of the KHDSS area.

Residents of the study area are predominantly Mijikenda, a Bantu grouping of nine tribes with Girima (45%), Chonyi (33%), and Kauma (11%) being the most common. About 55% of the population is considered absolutely poor with per capita monthly income of about 10 USD.

The majority (80%) depend on subsistence farming, which is constrained by the low agricultural potential of the land (only 19% is arable). Only 45% of people are literate. Crude birth and death rates for this area are approximately 35/1,000 and 6/1,000 per year, respectively, and migration rates are approximately 100/1,000 per year, most of which is within the study area (Scott et al., 2012).

### Cohort identification and follow‐up

We identified cohorts of people with and without ACE in a baseline cross‐sectional survey (Edwards et al., 2008). The survey was undertaken by door‐to‐door visits of all residences within the KHDSS between August and November 2003.

The three‐stage method used to identify cases has been described elsewhere in detail (Edwards et al., 2008; Ngugi et al., 2012, 2013). Briefly, the first stage (stage I, SI) involved asking the head of each household two screening questions to identify members of the household with a history of convulsions (Appendix S1). Those that were positive were followed in stage II (SII) and were interviewed by a team of field personnel trained to administer a more detailed and specific screening tool for epilepsy (Appendix S2) (Placencia et al., 1992). Respondents positive in SII were then evaluated in stage III (SIII), where a diagnosis of ACE was made using detailed clinical history taken by a clinician trained in epilepsy and fluent in the local languages. The International League Against Epilepsy (ILAE) definitions of active epilepsy were used (Commission on Epidemiology & Prognosis: International League Against Epilepsy, 1993; Meinardi et al., 2001; Thurman et al., 2011), but a cutoff of at least one seizure in the preceding 12 months was used for active epilepsy (Meinardi et al., 2001; Ministry of Health, Kenya, 2002; Thurman et al., 2011). We excluded children younger than 6 years of age because of difficulties in differentiating between neonatal seizures, febrile seizures, and epilepsy in this age group.

Deaths and migration were identified in the routine reenumeration within the KHDSS during the follow‐up period. In the second cross‐sectional survey, conducted between December 2007 and June 2008, we attempted to interview every subject in the cohort who had not died and was still a resident in the KHDSS (Ngugi et al., 2013). Due to logistical and cost implications, no attempt was made to find subjects from the first survey who had migrated out of the study area. The second cross‐sectional survey used the same methodology as the first cross‐sectional survey to identify new cases of ACE.

In both surveys, we defined ACE as at least two unprovoked convulsions (tonic and/or clonic seizures) of which one occurred in the 12 months preceding the survey (Edwards et al., 2008; Ngugi et al., 2013). An incident case of CE was a participant who was found not to have ACE in the first survey but was subsequently detected as a case in the follow‐up survey. For an incident case, the first seizure may have occurred days or years before the second survey (Jallon et al., 2001). These cases were included in the analysis because there had been no other diagnosis of epilepsy before the presentation in the current study (Hauser et al., 1996; Olafsson et al., 2005). We thus defined an incident case on the basis of the date of diagnosis of epilepsy rather than on the date of the second unprovoked seizure (Commission on Epidemiology & Prognosis: International League Against Epilepsy, 1993), since the recall of dates by the patients and their caretakers is often poor. Seizure types for all cases were classified by the study neurologist (CRN) using the recommendations of the Commission of the Classification and Terminology of the ILAE (Berg et al., 2010; Thurman et al., 2011).

### Analysis

We estimated incidence of CE as the number of new diagnoses of ACE among the subjects that did not have ACE in the baseline cross‐sectional survey, divided by the total person years of observation (pyo). All person‐observation time for those lost due to out‐migration or death was excluded from the denominator. We estimated an overall incidence together with 95% confidence intervals (95% CI) assuming a Poisson model for the number of incident cases. Given that some people may have developed epilepsy during the follow‐up but were inactive at the second survey, we assumed that the incidence estimated in this study was a minimum rate. Incidence rates were estimated by seizure type, age‐group, sex, and administrative location.

We used the technique of multiple imputations (MIs) with five imputations to reduce bias due to loss to follow‐up between stages of the two surveys (Rubin, 1987; van Buuren, 2007; Ngugi et al., 2013). The imputation model used the outcome from SI to impute SII where this was missing, and the outcome from SII to impute SIII. All incidence estimates were adjusted for the low sensitivity (48.6%) of the screening methodology (Ngugi et al., 2012). This was done for both the baseline and follow‐up prevalence surveys to preclude the inclusion of cases missed in the baseline as incident cases (thereby increasing the incidence) and to include new‐onset cases that may have been excluded at the follow‐up (leading to under estimation of incidence).

Incidence rates were compared between age (grouped as 6–12, 13–17, 18–28, 29–49, and 50+ year age bands), sex (female, male), ethnicity (Giriama, Chonyi, Kauma, Other Mijikenda, Luo, and other), and administrative area (Roka‐Matsangoni‐Mida, Ngerenya, Tezo, Chonyi, Sokoke, Jaribuni, Junju, Takaungu, and Kilifi Township) categories using incidence rate ratios obtained from Poisson regression models.

To determine if mortality in new‐onset epilepsy was a possible cause of underestimation of incidence, we compared the proportions of patients with the first seizure at the beginning of follow‐up (in 2003) to those who experienced the first seizure nearer the reevaluation survey (i.e., in 2007).

We further compared the age‐specific prevalence between the baseline (2003) and reevaluation survey (2008) to determine if the reduction in bacterial meningitis (Cowgill et al., 2006) and more lately malaria (O'Meara et al., 2008; Kariuki et al., 2011) in the last decade led to a corresponding reduction in the prevalence of epilepsy.

All analyses were carried out in STATA version 11 (StataCorp, College Station, TX, U.S.A.).

Written informed consent was obtained from all study participants. Where the patient was a child or an adult who could not respond, a caregiver was interviewed. Approval for the study was obtained from the Kenya Medical Research Institute/National Ethical Review Committee.

## Results

In the baseline survey, we identified 448 subjects with ACE and 150,960 subjects without ACE. At the follow‐up survey, 45,809 (30.3%) participants of the cohort could not be found within the KHDSS because 42,506 (92.8%) had out‐migrated, 2,703 (5.9%) had died, and 600 (1.3%) could not be traced. There were no significant differences in the baseline characteristics of cohort members who were identified in the 2008 survey compared to those not found (Table 1) as well as among cases of ACE identified at baseline that were lost to follow‐up and those that remained in the study area (Table S1).

**Table 1:** Main characteristics of the incidence study completers and those lost to follow‐up

Attribute	Category	Found in the KDHSS		Not found in the 2008 census	
		N	%	N	%
Sex	Male	47,548	45.2	22,278	48.6
Median age (IQR)		20 (11–38)		20 (14–30)	
Area	Roka‐M‐M^a^	14,691	14.0	5,654	12.3
	Ngerenya	6,352	6.0	2,703	5.9
	Tezo	13,590	12.9	5,843	12.8
	Chonyi	21,133	20.1	6,841	14.9
	Sokoke	3,886	3.7	1,691	3.7
	Jarubini‐Kauma	6,143	5.8	2,348	5.1
	Junju/Mtwapa	17,979	17.1	7,626	16.7
	Takaungu	10,723	10.2	4,454	9.7
	Kilifi Township	10,654	10.13	8,649	18.9
Ethnicity	Giriama	45,310	43.1	20,506	44.8
	Chonyi	36,687	34.9	12,757	27.9
	Kauma	12,133	11.5	4,872	10.6
	Other Mijikendas	5,723	5.4	3,002	6.6
	Luos	593	0.6	856	1.9
	Others	4,702	4.5	3,803	8.3
Stage I status at baseline	Positive	1,425	1.4	475	1.0
All cohort members		105,151		45,809	

a Roka‐Matsangoni‐Mida.

There was no significant difference in the crude age‐specific prevalence between the surveys conduct in 2003 and 2008 (Table 2). In particular there were no differences in the prevalence of the 6–12 years age group between the surveys.

**Table 2:** Comparison of unadjusted age‐specific prevalence of ACE between the baseline (2003) and reevaluation (2008) surveys

Age group	2003 survey				2008 Survey				Differences between the surveys p‐value
	Screened	Number of cases identified	Prevalence per 1000	95% CI	Screened	Number of cases identified	Prevalence per 1000	95% CI	
0–5	–	–	–	–	42,098	84	2.0	1.6–2.5	–
6–12	41,727	127	3.0	2.5–3.6	52,573	152	2.9	2.5–3.4	0.67
13–17	25,281	91	3.6	2.9–4.4	34,398	145	4.2	3.5–4.8	0.23
18–28	31,989	118	3.7	3.1–4.4	38,003	156	4.1	3.5–4.8	0.38
29–49	34,050	71	2.1	1.6–2.6	42,631	102	2.4	2.0–2.9	0.37
≥50	18,361	41	2.2	1.6–3.0	24,178	60	2.5	1.9–3.2	0.60
All ages	151,408	448	3.0	2.7–3.2	233,881	699	3.0	2.8–3.2	0.87

We identified 194 incident cases of CE. The median (interquartile range, IQR) age of incident cases was 19.5 (14–31) years, and 49% were male. We estimated that there would be 233 incident cases (after adjustment for loss to follow‐up) and 479 incident cases (after adjustment for both loss to follow‐up and sensitivity of the screening methodology). The total person‐years of observation (pyo) for this cohort was 623,004, after MIs with five rounds. The minimum crude incidence rate was 37.6/100,000 pyo (95% CI 32.7–33.3) and was 77.0/100,000 pyo (95% CI 67.7–87.4) after adjusting for loss to follow‐up and the sensitivity of the three‐stage survey methodology. At an adjusted rate of 51.6/100,000 pyo (95% CI 42.5–62.2), incidence of focal seizures was more than two times that of generalized seizures in this population, although a large proportion of convulsive seizures could not be classified according to onset due to lack of adequate information (Table 3).

**Table 3:** Incidence of convulsive seizure in Kilifi, Kenya

Seizure type	Unadjusted			Adjusted for attrition			Adjusted for attrition and sensitivity		
	Cases	Incidence	95% CI	Cases	Incidence	95% CI	Cases	Incidence	95% CI
Generalized	43	8.3	6–11.2	50	9.7	7.0–13.1	103	19.9	14.4–26.9
Generalized convulsive	41	8	5.9–10.8	48	9.3	6.9–12.6	98	19.2	14.12–25.9
Generalized other motor	2	0.4	0.1–1.6	2	0.5	0.1–1.9	5	1.0	0.2–3.8
Focal	111	21.5	17.7–25.9	130	25.1	20.7–30.2	267	51.6	42.5–62.2
Focal motor	53	10.2	7.4–14.8	62	11.9	8.6–17.3	127	24.5	17.8–35.5
Focal sec gen convulsive	58	11.2	8.7–14.6	68	13.1	10.2–17.0	139	26.9	20.89–35.1
Other motor undetermined	6	1.2	0.14–2.5	7	1.4	0.2–2.9	14	2.9	0.3–6.0
Other convulsive undetermined	34	6.6	4.6–9.2	40	7.7	5.4–10.7	82	15.9	11.0–22.1

There was strong evidence of variation of incidence of CE with age, with the highest incidence in the 6–17 year olds, lowest in 28–49 year olds, and an increase in the ≥50 year olds (Table 4). Incidence did not vary according to sex, administrative area of residence, or ethnicity (Table 4).

**Table 4:** Incidence rates and ratios for age, sex, ethnicity, and area of residence for convulsive epilepsy in Kilifi, Kenya

	Pyo^a^	Cases^a^	Incidence rate^a^	95% CI	Rate ratio	95% CI	p‐value
Age group							
6–12	197,040	189	96.1	78.4–117.9	1.0		0.008
13–17	90,246	82	91.2	66.9–124.3	0.91	0.62–1.33	
18–28	102,426	84	82.3	60.7–111.9	0.82	0.56–1.20	
29–49	148,020	56	37.4	25.7–54.7	0.46	0.31–0.71	
50+	85,272	68	79.6	56.6–111.9	0.86	0.57–1.28	
Sex							
Female	341,406	249	72.8	61.1–87.2	1.0		0.30
Male	281,598	230	81.9	67.9–98.6	1.2	0.9–1.5	
Ethnicity							
Giriama	268,206	210	78.2	64.4–95.1	1.0		0.65
Chonyi	218,370	158	72.6	58.0–90.7	0.94	0.69–1.27	
Kauma	71,814	68	94.7	67.3–132.9	1.35	0.92–1.98	
Other Mijikenda	33,864	29	85.0	50.4–143.6	0.98	0.54–1.78	
Luo	3,390	0	0	–	0	–	
Other	27,360	14	52.7	25.1–110.5	1.01	0.53–1.93	
Area							
Roka‐Matsangoni‐Mida	86,808	93	106.6	79.6–142.8	1.0		0.35
Ngerenya	37,962	29	75.9	44.9–128.2	0.65	0.35–1.20	
Tezo	80,664	56	68.9	47.3–100.4	0.61	0.38–.098	
Chonyi	126,204	93	73.5	54.7–98.4	0.63	0.41–0.95	
Sokoke	23,070	25	107.0	60.7–188.5	0.82	0.41–1.62	
Jaribuni	36,612	33	89.9	55.1–146.7	0.77	0.43–1.38	
Junju	106,938	80	75.1	54.7–102.7	0.67	0.44–1.03	
Takaungu	63,762	43	67.7	44.2–103.9	0.56	0.33–0.96	
Kilifi Township	60,984	29	47.3	28.0–79.8	0.59	0.34–1.00	

a Means estimated after adjustment for loss to follow‐up and sensitivity of the screening methodology.

Of the 194 incident cases detected in the 2008 prevalence survey, 130 experienced the first nonfebrile seizure prior to the baseline epilepsy survey of 2003, 64 had seizure onset during the follow‐up period (i.e., between 2003 and 2007), and in 10 the date of onset was not available. During follow‐up, the highest proportion of incident cases (31.3%) occurred in the year preceding the reevaluation survey (2007), whereas the lowest (12.5%) was in the baseline year (2003), and these proportions were significantly different (p = 0.008). However, there was no significant trend in the number of incident cases over the years (p‐value for trend = 0.18).

## Discussion

We used two cross‐sectional surveys 5 years apart within an existing HDSS of a rural population in Kenya to identify incident cases of ACE, and we estimated a minimum incident rate of convulsive epilepsy at 77.0/100,000 persons per year, which varied with age. Cases were identified in three‐stage cross‐sectional surveys at the beginning and at the end of the follow‐up period. ILAE definitions with modified timelines (Commission on Epidemiology & Prognosis: International League Against Epilepsy, 1993; Thurman et al., 2011) were used to define cases and noncases (Edwards et al., 2008; Ngugi et al., 2013).

Within the KHDSS framework, the study benefitted from continuous follow‐up for migration and death, but we were able to determine the cumulative incidence of new cases from the cross‐sectional study that was conducted at the end of the study. As such, we have reported a minimum incident rate, since we were not able to detect newly developed epilepsy cases that may have died shortly after the onset of seizures or been inactive during the reevaluation survey of 2008. For example, of the 448 people with ACE identified at the baseline survey of 2003, 61 had died as at 31 December 2011. About 10% died within the first 3 years and 23% within the first 5 years of onset of seizures (data not shown). In addition, it was not possible to determine the epilepsy status of residents who migrated out of the KHDSS or died, although the demographic profiles of the incidence cohort (as well as for the cases of ACE) identified at baseline were similar to those of the cohort members who were found at the second cross‐sectional survey. In particular, the proportions of people with histories of convulsions at baseline (i.e., positive in the first stage of the baseline survey and therefore more likely to develop epilepsy) were similar among those lost and those who remained in the cohort. We also used the MIs to determine and incorporate the proportion of incident cases among participants lost to follow‐up into the overall estimate of incidence, thereby minimizing selection bias. Because mortality is typically highest in the first 1–2 years following the onset of seizures (Cockerell et al., 1994; Forsgen et al., 2005), incidence may have been underestimated as we evaluated the cohort 5 years apart. This is supported by the timing of the first seizure, which showed a significantly higher proportion of new‐onset epilepsy nearer to the reevaluation survey compared to that immediately after the baseline survey.

We estimated the incidence rate of CE at 77/100,000 pyo. Prevalence studies in LMICs that have included nonconvulsive epilepsies (Kaamugisha & Feksi, 1988; Birbeck & Kalichi, 2004) have reported prevalence estimates that were 2–3 times higher than those that studied convulsive epilepsies only (Rwiza et al., 1992; Tekle‐Haimanot et al., 1997). Assuming similar rates for mortality and remission across studies, these findings would suggest that the incidence of all epilepsies in our population could be in the range of 154–231/100,000 persons per year, which is higher than rates reported for all epilepsies from other studies in LMICs. These estimates have varied widely, from 49/100,000 in India (Mani et al., 1998), 69.4/100,000 in Benin (Houinato et al., 2013), 73/100,000 in Tanzania (Rwiza et al., 1992), and 92/100,000 in Honduras (Medina et al., 2005). Our extrapolated incidence rates for all epilepsies would, however, be similar to the rate of 215/100,000 reported in an Onchocerciasis endemic area of Uganda (Kaiser et al., 1998), the 162/100,000 reported in an Cysticercosis endemic region of Peru (Villaran et al., 2009), and within the range estimated for LMICs in a recent review of incidence of life‐time epilepsy (Ngugi et al., 2011). These data suggest that incidence is likely to vary depending on the geographic distribution and risk factors associated with epilepsy, although these are unadjusted estimates and therefore the comparisons should be interpreted with caution.

The observation that incidence of focal seizures was more than twice that of generalized seizures could suggest that factors such as neonatal insults and infections/infestations of the central nervous system, which can be prevented, are more important etiologies of convulsive epilepsy in this area (Ngugi et al., 2013).

Compared to other studies that have used a 5‐year cutoff for the last seizure to define active epilepsy (Commission on Epidemiology & Prognosis: International League Against Epilepsy, 1993), we used a definition in which the last seizure was within the previous 12 months in our baseline and reevaluation prevalence surveys, as suggested by the ILAE for studies in the LMICs (Meinardi et al., 2001; Thurman et al., 2011) and because recall of seizures over a year ago is poor (Newton CR, Mung'alla‐Odera V, unpublished data). Therefore, under the ILAE definition, the incidence of convulsive epilepsy in this population could be considerably higher. Poor recall of seizures and early mortality were still likely to reduce the incidence estimates. For instance, if a respondent experienced the last seizure in the preceding 12 months but could not recall earlier seizures if they occurred long ago they were likely to be misclassified as a case of single seizure. These two factors (poor recall and early mortality) could explain the significant differences in the proportions of onset by years.

The age‐incidence relationship in our study showed a U‐shaped curve, with highest incidence in the children and adolescents and old aged adults, and lowest in 28–49 year olds, as has been observed in the high income countries (HICs) (Jallon et al., 1997; Olafsson et al., 2005). In the 6–28 year age group, onset of recurrent seizures could be related to perinatal insults and infections of the central nervous system, particularly in the younger children (Preux & Druet‐Cabanc, 2005; Ngugi et al., 2013). Many genetically determined epilepsies may also manifest during this age (Goudsmit & van der Waals, 1983; Edwards et al., 2008). In the adolescents and young adults, recurrent seizures may develop as a consequence of temporally remote etiologies that result in static encephalopathies, for example, infectious brain insults earlier in life, cerebral palsy, and head injuries (Ogunniyi et al., 1987; Edwards et al., 2008). However, there was no change in the prevalence between the surveys in 2003 and 2008, particularly in the 6–12 year age group, despite the introduction of Haemophilus influenza type B (*Hib*) vaccine in 2001 (Cowgill et al., 2006), and a reduction of the incidence of severe malaria (O'Meara et al., 2008) and acute seizures (Kariuki et al., 2011) admitted to Kilifi District Hospital since 2004. These potential causes may not be responsible for a significant population‐attributable fraction of ACE, but could cause other forms on nonconvulsive epilepsies (Pomeroy et al., 1990), or there may not have been sufficient time for the reduction in the incidence of these conditions to reflect in the decrease in the prevalence. The incidence rate was not different between males and females in our study population.

The higher incidence rate in the elderly (50+ years) compared to younger adults has also been observed in industrialized countries (Sander et al., 1990) and in one study in an LMIC (Rwiza et al., 1992) but not in another LMIC (Tekle‐Haimanot et al., 1997). Although it has not been documented in LMICs, elderly people may experience higher risk of developing epilepsy associated with progressive brain conditions that include tumors, cerebrovascular accidents and neurodegenerative conditions such as Alzheimer's disease (Commission on Epidemiology & Prognosis: International League Against Epilepsy, 1993).

In view of the high incidence of CE in this population, etiologic studies are necessary, preferably in incident cases in order to elucidate causes, particularly among children, adolescents, and the elderly who are at highest risk. Neuroimaging may also help elucidate the causes. Furthermore, these age groups should be targeted for preventive public health interventions.

## Limitations of the Study

We utilized a three‐stage survey methodology that had low sensitivity (48.6%) to detect cases of ACE. We were able to adjust estimates for the low sensitivity, but it is possible that some cases were missed during the baseline survey and counted as incident cases in the follow‐up survey resulting in an overestimation of the incidence rate. Furthermore, poor recall of seizures, particularly those that occurred long ago, could have led to underestimation of incidence.

Premature mortality due to epilepsy, remission, as well as lack of identification of newly onset epilepsy cases that may have been inactive during the reevaluation survey (2008) may have led to an underestimate of the number of incident cases. Future studies would benefit from a more frequent reevaluation (e.g., every 6 months) of the cohort to increase the detection of incident cases. We also excluded children ≤5 years of age from this study, which may imply that the incidence of epilepsy in the study area could be considerably higher given that most of the epilepsy starts during early childhood.

## Conclusion

We estimated the incidence of CE in a large population‐based prospective cohort study that was twice as high as that in HICs and within range of estimates reported for LMICs. Incidence of epilepsy was highest in children, adolescents, and the elderly; the causes should be investigated to prevent the development of epilepsy in these age groups. The incidence of focal epilepsy was more than twice that of generalized epilepsy, suggesting that etiologies that can be prevented could be most important in this area.

## Disclosure

All authors confirm that they have no financial or personal interest, including advisory board affiliation, in any company or organization sponsoring the research. We confirm that we have read the Journal's position on issues involved in ethical publication and affirm that this report is consistent with those guidelines.

## Supplementary Material

**Table S1.** Main characteristics of epilepsy cases that completed the study and those lost to follow‐up.

**Appendix S1. **Stage I (SI) of the cross‐sectional survey: (Census screen for convulsions).
**Appendix S2. **Stage II (SII) screening questions.

## References

[epi12236-bib-0001] Berg AT, Berkovic SF, Brodie MJ, Buchhalter J, Cross JH, Emde V, Boas W, Engel J, French J, Glauser TA, Mathern GW, Moshe SL, Nordli D, Plouin P, Scheffer IE. (2010) Revised terminology and concepts for organization of seizures and epilepsies: report of the ILAE Commission on Classification and Terminology, 2005–2009. Epilepsia 51:676–685 20196795 10.1111/j.1528-1167.2010.02522.x

[epi12236-bib-0002] Birbeck GL, Kalichi EM. (2004) Epilepsy prevalence in rural Zambia: a door‐to‐door survey. Trop Med Int Health 9:92–95 14728612 10.1046/j.1365-3156.2003.01149.x

[epi12236-bib-0105] Carpio A, Bharucha NE, Jallon P, Beghi E, Campostrini R, Zorzetto S, Mounkoro PP. (2005) Mortality of epilepsy in developing countries. Epilepsia 46(Suppl. 11):28–32 16393175 10.1111/j.1528-1167.2005.00404.x

[epi12236-bib-0003] Cockerell OC, Johnson AL, Sander JW, Hart YM, Goodridge DM, Shorvon SD. (1994) Mortality from epilepsy: results from a prospective population‐based study. Lancet 344:918–921 7934347 10.1016/s0140-6736(94)92270-5

[epi12236-bib-0004] Commission on Epidemiology and Prognosis: International League Against Epilepsy . (1993) Guidelines for epidemiologic studies on epilepsy. Epilepsia 34:592–596 8330566 10.1111/j.1528-1157.1993.tb00433.x

[epi12236-bib-0005] Cowgill KD, Ndiritu M, Nyiro J, Slack MP, Chiphatsi S, Ismail A, Kamau T, Mwangi I, English M, Newton CR, Feikin DR, Scott JA. (2006) Effectiveness of Haemophilus influenzae type b Conjugate vaccine introduction into routine childhood immunization in Kenya. JAMA 296:671–678 16896110 10.1001/jama.296.6.671 PMC1592684

[epi12236-bib-0106] Diop AG, Hesdorffer DC, Logroscino G, Hauser WA. (2005) Epilepsy and mortality in Africa: A review of the literature. Epilepsia 46(Suppl. 11):33–35 16393176 10.1111/j.1528-1167.2005.00405.x

[epi12236-bib-0006] Edwards T, Scott AG, Munyoki GV, Mungalla‐Odera V, Chengo E, Bauni E, Kwasa T, Sander JWAS, Neville BG, Newton CR. (2008) Active convulsive epilepsy in a rural district of Kenya: A study of prevalence and possible risk factors. Lancet Neurol 7:50–56 18068520 10.1016/S1474-4422(07)70292-2 PMC4058896

[epi12236-bib-0007] Forsgen L, Hauser W, Olafsson E, Sander JW, Salinpaa M, Tomson T. (2005) Mortality and epilepsy in developed countries: a review. Epilepsia 46:18–27 16393174 10.1111/j.1528-1167.2005.00403.x

[epi12236-bib-0008] Goudsmit J, van der Waals FW. (1983) Endemic epilepsy in an isolated region of Liberia. Lancet 1:528–529 6131234 10.1016/s0140-6736(83)92215-8

[epi12236-bib-0009] Hauser E, Freilinger M, Seidl R, Groh C. (1996) Prognosis of childhood epilepsy in newly referred patients. J Child Neurol 11:201–204 8734022 10.1177/088307389601100307

[epi12236-bib-0010] Houinato D, Yemadje LP, Glitho G, Adjien C, Avode G, Druet‐Cabanac M, Preux PM. (2013) Epidemiology of epilepsy in rural Benin: prevalence, incidence, mortality, and follow‐up. Epilepsia 54:757–763 23350750 10.1111/epi.12082

[epi12236-bib-0011] Jallon P, Goumaz M, Haenggeli C, Morabia A. (1997) Incidence of first epileptic seizures in the canton of Geneva, Switzerland. Epilepsia 38:547–552 9184600 10.1111/j.1528-1157.1997.tb01139.x

[epi12236-bib-0012] Jallon P, Loiseau P, Loiseau J. (2001) Newly diagnosed unprovoked epileptic seizures: presentation at diagnosis in CAROLE study. Coordination Active du Reseau Observatoire Longitudinal de l' Epilepsie. Epilepsia 42:464–475 11440341 10.1046/j.1528-1157.2001.31400.x

[epi12236-bib-0013] Kaamugisha J, Feksi AT. (1988) Determining the prevalence of epilepsy in the semi‐urban population of Nakuru, Kenya, comparing two independent methods not apparently used before in epilepsy studies. Neuroepidemiology 7:115–121 3136404 10.1159/000110144

[epi12236-bib-0014] Kaiser C, Asaba G, Leichsenring M, Kabagambe G. (1998) High incidence of epilepsy related to onchocerciasis in West Uganda. Epilepsy Res 30:247–251 9657652 10.1016/s0920-1211(98)00007-2

[epi12236-bib-0100] Kaiser C, Asaba G, Kasoro S, Rubaale T, Kabagambe G, Mbabazi M. (2007) Mortality from epilepsy in an onchocerciasis‐endemic area in West Uganda. Trans R Soc Trop Med Hyg 101:48–55 16905166 10.1016/j.trstmh.2006.06.003

[epi12236-bib-0015] Kariuki SM, Ikumi M, Ojal J, Sadarangani M, Idro R, Olotu A, Bejon P, Berkley JA, Marsh K, Newton CR. (2011) Acute seizures attributable to falciparum malaria in an endemic area on the Kenyan coast. Brain 134:1519–1528 21482551 10.1093/brain/awr051 PMC3097888

[epi12236-bib-0102] Kwan P, Sander JW. (2004) The natural history of epilepsy: an epidemiological view. J Neurol Neurosurg Psychiatry 75:1376–1381 15377680 10.1136/jnnp.2004.045690 PMC1738749

[epi12236-bib-0101] Leonardi M, Ustan TB. (2002) The global burden of epilepsy. Epilepsia 43:21–25 12190974 10.1046/j.1528-1157.43.s.6.11.x

[epi12236-bib-0016] Mani KS, Rangan G, Srinivas HV, Kalyanasundaram S, Narendran S, Reddy AK. (1998) The Yelandur study: a community‐based approach to epilepsy in rural South India‐ epidemiological aspects. Seizure 7:281–288 9733402 10.1016/s1059-1311(98)80019-8

[epi12236-bib-0017] Medina MT, Duron RM, Martinez L, Osorio JR, Estrada AL, Zuniga C, Cartagena D, Collins JS, Holden KR. (2005) Prevalence, incidence, and etiology of epilepsies in rural Honduras: the Salama Study. Epilepsia 46:124–231 15660778 10.1111/j.0013-9580.2005.11704.x

[epi12236-bib-0018] Meinardi H, Scott RA, Reis R, Sander JW. (2001) The treatment gap in epilepsy: the current situation and ways forward. Epilepsia 42:136–149 11207798 10.1046/j.1528-1157.2001.32800.x

[epi12236-bib-0019] Ministry of Health, Kenya . (2002) Central nervous system In Kimathi NA, Macheni JN, Muriithi A (Eds) Clinical guidelines. 2nd ed Ministry of Health, Govenrment of Kenya, Nairobi, pp. 55–60

[epi12236-bib-0103] Newton CR, Garcia H. (2012) Epilepsy in poor regions of the world. Lancet 380:1193–1201 23021288 10.1016/S0140-6736(12)61381-6

[epi12236-bib-0104] Ngugi AK, Bottomley C, Kleinschmidt I, Sander JW, Newton CR. (2010) Estimation of the burden of active and lifetime epilepsy: a meta‐analytic approach. Epilepsia 51:883–890 20067507 10.1111/j.1528-1167.2009.02481.x PMC3410521

[epi12236-bib-0020] Ngugi AK, Kariuki SM, Bottomley C, Kleinschmidt I, Sander JW, Newton CR. (2011) Incidence of epilepsy: a systematic review and meta‐analysis. Neurology 77:1005–1012 21893672 10.1212/WNL.0b013e31822cfc90 PMC3171955

[epi12236-bib-0021] Ngugi AK, Bottomley C, Chengo E, Kombe MZ, Kazungu M, Bauni E, Mbuba CK, Kleinschmidt I, Newton CR. (2012) The validation of a three‐stage survey methodology for detecting active convulsive epilepsy in demographic surveillance systems. Emerg Themes Epidemiol 9:8 23171721 10.1186/1742-7622-9-8 PMC3549939

[epi12236-bib-0022] Ngugi AK, Bottomley C, Kleinschmidt I, Wagner RG, Kakooza‐Mwesige A, Ae‐Ngibise K, Owusu‐Agyei S, Masanja H, Kamuyu G, Odhiambo R, Chengo E, Sander JW, Newton CR. (2013) Prevalence of active convulsive epilepsy and associated risk factors in sub‐Saharan Africa: cross‐sectional and case control studies. Lancet Neurol 12:253–263 23375964 10.1016/S1474-4422(13)70003-6 PMC3581814

[epi12236-bib-0023] Ogunniyi A, Osuntokun BO, Bademosi O, Adeuja AO, Schoenberg BS. (1987) Risk factors for epilepsy: case‐control study in Nigerians. Epilepsia 28:280–285 3582292 10.1111/j.1528-1157.1987.tb04219.x

[epi12236-bib-0024] Olafsson E, Ludvigsson P, Gudmundsson G, Hesdorffer D, Kjartansson O, Hauser WA. (2005) Incidence of unprovoked seizures and epilepsy in Iceland and assessment of the epilepsy syndrome classification: a prospective study. Lancet Neurol 4:627–634 16168931 10.1016/S1474-4422(05)70172-1

[epi12236-bib-0025] O'Meara WP, Bejon P, Mwangi TW, Okiro EA, Peshu N, Snow RW, Newton CR, Marsh K. (2008) Effect of a fall in malaria transmission on morbidity and mortality in Kilifi, Kenya. Lancet 372:1555–1562 18984188 10.1016/S0140-6736(08)61655-4 PMC2607008

[epi12236-bib-0026] Placencia M, Sander JW, Shorvon SD, Ellison RH, Cascante SM. (1992) Validation of a screening questionnaire for the detection of epileptic seizures in epidemiological studies. Brain 115:783–794 1628202 10.1093/brain/115.3.783

[epi12236-bib-0027] Pomeroy SL, Holmes SJ, Dodge PR, Feigin RD. (1990) Seizures and other neurologic sequelae of bacterial meningitis in children. N Engl J Med 323:1651–1657 2233962 10.1056/NEJM199012133232402

[epi12236-bib-0028] Preux PM, Druet‐Cabanc M. (2005) Epidemiology and aetiology of epilepsy in sub‐Saharan Africa. Lancet Neurol 4:21–31 15620854 10.1016/S1474-4422(04)00963-9

[epi12236-bib-0029] Rubin DB. (1987) Multiple imputation for nonresponse in surveys. Wiley, New York

[epi12236-bib-0030] Rwiza HT, Kilonzo GP, Haule J, Matuja WB, Mteza I, Mbena P, Kilima PM, Mwaluko G, Mwang'ombola R, Mwaijande F. (1992) Prevalence and incidence of epilepsy in Ulanga, a rural Tanzanian district: a community‐based study. Epilepsia 33:1051–1056 1464263 10.1111/j.1528-1157.1992.tb01758.x

[epi12236-bib-0107] Sander JW. (1993) Some aspects of prognosis in the epilepsies: A review. Epilepsia 34:1007–1016 8243349 10.1111/j.1528-1157.1993.tb02126.x

[epi12236-bib-0031] Sander JW, Hart YM, Johnson AL, Shorvon SD. (1990) National General Practice Study of Epilepsy: newly diagnosed epileptic seizures in a general population. Lancet 336:1267–1271 1978113 10.1016/0140-6736(90)92959-l

[epi12236-bib-0032] Scott JA, Bauni E, Moisi JC, Ojal J, Gatakaa H, Nyundo C, Molyneux CS, Kombe F, Tsofa B, Marsh K, Peshu N, Williams TN. (2012) Profile: the Kilifi Health and Demographic Surveillance System (KHDSS). Int J Epidemiol 41:650–657 22544844 10.1093/ije/dys062 PMC3396317

[epi12236-bib-0108] Sillanpaa M, Camfield PR, Camfield CS, Aromaa M, Helenius H, Rautava P, Hauser WA. (2008) Inconsistency between prospectively and retrospectively reported febrile seizures. Dev Med Child Neurol 50:25–28 18173625 10.1111/j.1469-8749.2007.02006.x

[epi12236-bib-0033] Tekle‐Haimanot R, Forsgren L, Ekstedt J. (1997) Incidence of epilepsy in rural central Ethiopia. Epilepsia 38:541–546 9184599 10.1111/j.1528-1157.1997.tb01138.x

[epi12236-bib-0034] Thurman DJ, Beghi E, Begley CE, Berg AT, Buchhalter JR, Ding D, Hesdorffer DC, Hauser WA, Kazis L, Kobau R, Kroner B, Labiner D, Liow K, Logroscino G, Medina MT, Newton CR, Parko K, Paschal A, Preux PM, Sander JW, Selassie A, Theodore W, Tomson T, Wiebe S. (2011) Standards for epidemiologic studies and surveillance of epilepsy. Epilepsia 52(Suppl. 7):2–26 21899536 10.1111/j.1528-1167.2011.03121.x

[epi12236-bib-0035] van Buuren S. (2007) Multiple imputation of discrete and continuous data by fully conditional specification. Stat Methods Med Res 16:219–242 17621469 10.1177/0962280206074463

[epi12236-bib-0036] Villaran MV, Montano SM, Gonzalvez G, Moyano LM, Chero JC, Rodriguez S, Gonzalez AE, Pan W, Tsang VC, Gilman RH, Garcia HH. (2009) Epilepsy and neurocysticercosis: an incidence study in a Peruvian rural population. Neuroepidemiology 33:25–31 19325247 10.1159/000210019 PMC2826439

