# Supplementary material for: Incidence of convulsive epilepsy in a rural area in Kenya
Source: Epilepsia. 2013 Jun 10;54(8):1352–9. doi: 10.1111/epi.12236 (PMC4114531; doi:10.1111/epi.12236)
Supplement: Supplementary file 1 — Table S1. Main characteristics of epilepsy cases that completed the study and those lost to follow‐up. [file epi-54-1352-s1.doc]

Supplementary table 1: Main characteristics of epilepsy cases that completed the study and those lost to follow-up.

|  |  | **Found in the KDHSS** | | **Not found on the 2008 census** | |
| --- | --- | --- | --- | --- | --- |
| **Attribute** | **Category** | **N** | **%** | **N** | **%** |
| **Sex** | Male | 174 | 51.8 | 53 | 47.3 |
| **Median age (IQR)** |  | 18.0 (11.5 – 29.0) |  | 18.0 (13.5 – 26.0) |  |
| **Area** | Roka-M-M† | 58 | 17.3 | 15 | 13.4 |
|  | Ngerenya | 38 | 11.3 | 11 | 9.8 |
|  | Tezo | 60 | 17.9 | 17 | 15.2 |
|  | Chonyi | 72 | 21.4 | 24 | 21.4 |
|  | Sokoke | 9 | 2.7 | 3 | 2.7 |
|  | Jarubini-Kauma | 24 | 7.1 | 11 | 9.8 |
|  | Junju/Mtwapa | 39 | 11.6 | 14 | 12.5 |
|  | Takaungu | 33 | 9.8 | 14 | 12.5 |
|  | Kilifi Township | 3 | 0.9 | 3 | 2.7 |
| **Ethnicity** | Giriama | 148 | 44.1 | 55 | 49.1 |
|  | Chonyi | 134 | 39.9 | 36 | 32.1 |
|  | Kauma | 39 | 11.6 | 13 | 11.6 |
|  | Other Mijikendas | 11 | 3.3 | 7 | 6.3 |
|  | Others | 4 | 1.2 | 0 | 0.0 |
|  | Luos | 0 | 0.0 | 1 | 0.9 |
| **All cases** |  | 336 | 75.0 | 112 | 25.0 |
