# Supplementary material for: Incidence of convulsive epilepsy in a rural area in Kenya
Source: Epilepsia. 2013 Jun 10;54(8):1352–9. doi: 10.1111/epi.12236 (PMC4114531; doi:10.1111/epi.12236)
Supplement: Supplementary file 2 — Appendix S1. Stage I (SI) of the cross‐sectional survey: (Census screen for convulsions). Appendix S2. Stage II (SII) screening questions. [file epi-54-1352-s2.doc]

## Appendix S1: Stage I (SI) of the cross-sectional survey: (Census screen for convulsions)

Q1: Do you have fits or has someone ever told you that you have fits?

Q2: Do you experience episodes in which your legs or arms have jerking movements or fall to the ground and lose consciousness?

## Appendix S2: Stage II (SII) screening questions

Q1. Have you ever had a fit?

Q2. Has someone ever told you that you have fits?

Q3. Have you ever been told that you have epilepsy or epileptic fits?

Q4. Have you ever had attacks in which you fall to the ground with loss of consciousness?

Q5. Have you ever fallen to the ground without a reason and experienced:

1. Twitching?
2. Shaking of the arms or legs without control?
3. Wetting yourself?
4. Biting of the tongue?

Q6. Have you ever been told by a doctor that you have epilepsy or epileptic fits?
